# Do we need to accurately perceive our heartbeats? Cardioceptive accuracy and sensibility are independent from indicators of negative affectivity, body awareness, body image dissatisfaction, and alexithymia

**DOI:** 10.1371/journal.pone.0287898

**Published:** 2023-07-05

**Authors:** János Körmendi, Eszter Ferentzi, Tara Petzke, Vera Gál, Ferenc Köteles

**Affiliations:** 1 Doctoral School of Psychology, ELTE Eötvös Loránd University, Budapest, Hungary; 2 Institute of Health Promotion and Sport Sciences, ELTE Eötvös Loránd University, Budapest, Hungary; 3 Ádám György Psychophysiology Research Group, Budapest, Hungary; 4 Psychological Institute, Johannes Gutenberg University, Mainz, Germany; 5 Károli Gáspár University of the Reformed Church in Hungary, Budapest, Hungary; Anglia Ruskin University, UNITED KINGDOM

## Abstract

Assessment of the acuity of heartbeat perception, dubbed cardioceptive accuracy, as well as its association with various psychological characteristics are hot topics of interoception research. In this study, we aimed (1) to replicate previously reported findings on the association between the mental tracking task and a novel motor tracking task that eliminates disturbing tactile sensations; and (2) to explore associations between performance in the latter task and indicators of negative affectivity (anxiety, depression, anxiety sensitivity, somatic symptom distress), alexithymia, body focus, and dissatisfaction with body image. 102 young people (age = 20.8±5.08 yrs) participated in the study. Mental tracking score was significantly higher than motor tracking scores, although they were strongly associated. Frequentist correlation analysis showed no significant associations between indicators of cardioceptive accuracy and questionnaire scores; Bayesian analysis indicated the lack of association for the majority of the cases. Similarly, detectors and non-detectors showed no differences in any of the assessed characteristics and Bayesian results typically supported the lack of associations. In conclusion, cardioceptive accuracy, as assessed with different tracking methods, is not associated with the aforementioned self-reported characteristics in young individuals.

## Introduction

The acuity of perception of heartbeat, that is, cardioceptive accuracy (CAc), is the most frequently assessed aspect of interoception [1–3]. The measurement of CAc, particularly in the case of the so-called mental tracking task or Schandry task [4, 5], is highly problematic as top-down factors, such as expectancy and knowledge on heart rate (HR), appear to play a substantial role in the process [6–14]. Measurement-related issues led to the development of new paradigms in this field, relying on signal detection theory [15], the Bayesian approach [16], or motor tracking instead of internal counting [17, 18]. Along with more strict instructions that explicitly prohibit estimation of heartbeats [19, 20], these paradigms attempt to reduce the top-down component of the measures of cardioception. However, cardiac perception necessarily encompasses top-down processes (1); thus, their impact can be limited to some extent but cannot be completely eliminated.

One feature that makes mental tracking measurements particularly malleable to top-down influences is that the correspondence between actual and counted heartbeats cannot be calculated; those showing high performance in the task might be counting something else than actual heartbeats, such as vague body sensations evoked by an internal rhythm [1, 21]. Motor tracking paradigms that record the timing of motor responses allow for such calculations–thus, only responses that are in synchrony with cardiac events are taken into consideration [17, 22]. In turn, sensory input generated by button presses might interfere with the perception of cardiac events [8, 23], possibly decreasing CAc. To minimize this impact, we developed a new motor tracking paradigm [18], which relies on finger movements (tracked with electromyography, EMG) instead of button presses. This way, only the proprioceptive component of the motor response remains a possibly disturbing factor of cardiac perception. CAc as assessed with this novel motor tracking paradigm was substantially lower than CAc based on mental counting (M±SD: 0.10±0.10 and 0.38±0.28, respectively). However, the association between the two indices was surprisingly strong (*r*_*S*_ = .81, *p* < .001). In addition, detectors’ (i.e., individuals showing significantly better than random performance in the motor tracking task) Schandry score was significantly higher than non-detectors’ score, which might indicate that validity of the Schandry task is not as poor as supposed by many authors. The first goal of the present study was the replication of these findings.

The assumption that cardioceptive accuracy, often considered a proxy measure of interoceptive accuracy, contributes to mental health is generally accepted in the literature [24, 25]; certain authors even propose that it can be the general factor behind psychopathology [26–28]. As the Schandry task represents a comparatively quick and simple paradigm, it was used in the vast majority of studies exploring the associations between cardioception and various mental conditions, such as different aspects of anxiety, depression, symptom reporting, body image, body awareness, and alexithymia. With respect to anxiety, Pollatos et al. [29] reported a significant positive association between trait anxiety and Schandry score. Similarly, Ehlers et al. [30] found a higher performance in panic patients as opposed to healthy controls. These findings were confirmed by an early meta-analysis [31], however, more recent meta-analyses reported no associations [32, 33]. Anxiety sensitivity, defined as fear of the negative consequences of anxiety, showed a positive association with CAc [31]. For depression, results are mostly inconclusive [33–35], although Pollatos and colleagues [36] found a negative correlation between Schandry score and depression. Findings of another recent meta-analysis suggest that those with moderate levels of depression show worse heartbeat perception than healthy controls, whereas there is no difference for severe depression [37]. Symptom reporting was associated with reporting bias but not with CAc in patients with somatic symptom disorder and illness anxiety disorder in a meta-analysis [38]. For groups without these pathologies, lower cardioceptive accuracy, as assessed with the Schandry task, was not significantly associated with higher levels of somatic symptom distress [15]. In this study, however, sensitivity (d’) in a novel CAc task showed a moderate negative association with symptom reporting. It was also proposed that body image concerns would be associated by worse interoceptive accuracy [39]. This idea was supported by a number of empirical findings. For example, body image dissatisfaction was reversely associated with cardioceptive performance [40, 41]. In the same vein, persons with anorexia nervosa–a condition highly associated with body dissatisfaction–showed decreased CAc compared to healthy controls [42]. In contrast, Drew et al. [43] were not able to find an association between Schandry task performance and body dissatisfaction, whereas Lutz et al. [44] reported a trend towards better CAc in anorexia nervosa. The lack of significant associations between body awareness and CAc were reported in several studies [41, 43, 45, 46]. Lastly, an important transdiagnostic factor for a large variety of psychopathological conditions is alexithymia, characterized by deficits in the abilities to identify and describe one’s feelings [47]. Following this definition, it makes sense to assume a link between alexithymia and low CAc [47–50]. A wide range of studies has shown a reverse association between alexithymia and performance on the Schandry task [47, 51–53]. Interestingly, improving CAc with a mental training led to changes in alexithymia [51]. In the study of Nicholson et al. [54], however, alexithymia was unrelated to cardioceptive accuracy.

The aforementioned validity issues with the Schandry task rendered the interpretation of this large body of empirical findings uncertain. If performance in the task is primarily determined by non-interoceptive processes, the reported associations with all these phenomena do not refer to a relationship with CAc. However, if the bottom-up (i.e. cardiac) component of the Schandry task is substantial, as suggested by our previous study [18] as well as by other studies [15, 55], the reported associations might exist and indicate the contribution of cardiac perception (as assessed with tracking tasks) to psychological functioning. The second goal of the study was the replication of the reported associations using both mental and motor tracking tasks.

## Methods

### Participants

*A priori* sample size calculation for a moderate positive association (r = 0.3, one-tailed; α = 0.05, β = 0.9) was conducted with the G*Power v3.1.9.4. software [56]. It resulted in a minimum sample size of N = 91. Our sample consisted of 102 young individuals (13 male; M_age_ = 20.8 yrs; SD_age_ = 5.08 yrs), with normal body fat level (M = 29.5%, SD = 8.61%) and blood pressure values (M_systolic_ = 116.3 Hgmm, SD_systolic_ = 12.0 Hgmm; M_diastolic_ = 73.0 Hgmm, SD_diastolic_ = 9.0 Hgmm). Participants were undergraduate university students who received partial credit for their participation. The study was approved by the Ethics Board of the Faculty; all participants read and signed an informed consent form before starting the measurement.

### ECG and EMG measurement

Physiological measurements were conducted using the NeXus system (NeXus Wireless Physiological Monitoring and Feedback: NeXus-10 Mark II, Version 1.02; BioTrace + Software for NeXus-10 Version: V201581; Mind Media BV, Herten, the Netherlands). Sampling rate was 1024 Hz. Cardiac activity was recorded with the modified Lead II design; electrodes were placed on the left and right collarbone and the right lower costal arch. EMG was recorded between two electrodes placed on the palmar side of the right forearm over the belly of the flexor digitorum superficialis muscle. Participants’ forearm was lying on their thigh in a relaxed position with the palm facing upward during the measurement. Identification of R-R peaks (ECG) and finger movements (EMG) was conducted with custom algorithms implemented in Matlab (Version: R2016a; The MathWorks, Inc., Natick, MA, USA) and Java (SE 19). The outcome of the algorithms was checked by visual inspection for all cases.

### Mental tracking task

Assessment of heartbeat perception was conducted in a seated position. Participants were asked to count their heartbeats silently during three randomly presented intervals (25, 35 and 50 seconds) after a 15 sec long practice phase. The counting started with a verbal NOW signal and stopped by a STOP signal, after which participants reported the number of heartbeats they counted. Participants were explicitly encouraged to say zero if they did not feel any heartbeats, but also encouraged to count if they had a slight sensation only (for the exact instruction, see S1 File). Individual scores were calculated for each session with the following formula: 1 - |(HB_recorded_—HB_counted_)/ HB_recorded_ |. Heartbeat perception score (CAc_mental_) was the average of the three measured scores. Cronbach’s alpha coefficient for the three trials of the Schandry task was .95.

After each trial, participants verbally rated their perceived performance (“*What do you think how many percent of your heartbeats were sensed*”) on a 100-point scale with the anchor points of “*0%*” and “*100%*”. Average of the three ratings (CSb_mental_) was considered an indicator of cardioceptive sensibility, i.e., confidence rating (2); its Cronbach’s alpha value was .94.

### Motor tracking task

In the motor tracking task, participants moved their index finger in response to sensed heartbeats instead of internal counting. The setting and the instruction were similar to those of the mental tracking task (for the instruction, see S1 File). Similar to the mental tracking task, mean of self-rated performance in the three trials (CSb_motor_) of the task was regarded as an indicator of cardioceptive sensibility; its Cronbach’s alpha value was 0.90.

Three measures of CAc were calculated in the motor tracking task. Beyond the formula used for the Schandry task, including all motor responses (CAc_motor_all_), another index (CAc_motor_acceptable_) included only those movements whose timing was in the acceptable time frame, that is, from 350 to 650 ms from the preceding R-peak (for a detailed description of the background, see [18]). In the case of multiple responses, only the first response was accepted. Cronbach’s alpha for both indices was .95. Finally, detectors and non-detectors were identified based on the distribution of motor responses relative to that of heartbeats. We applied circular statistics—the temporal distance of motor responses from the preceding R-peak was converted to angles in degrees. The average R-R distance of the entire time interval was regarded as 360 degrees. The dispersion of the angles was calculated as the vectorial average of the unit vectors at the angle with x-axis (horizontal axis) in the two-dimensional plane, called mean resultant vector [57]. The length of the mean resultant vector is between 0 and 1; values close to zero reflects high variability of angles, whereas values close to 1 indicate less variability, meaning higher level of consistency. The length of the mean resultant vector was considered the fourth indicator of CAc. Individuals with a significant (*p* < .05) deviation from random responses were considered detectors, whereas those with a non-significant distribution were regarded as non-detectors. In other words, we considered those individuals as detectors whose motor responses were significantly associated with the preceding cardiac events.

### Questionnaires

#### Toronto Alexithymia Scale (TAS-20)

As a measure of alexithymia, we used Bagby, Parker, and Taylor’s [58] TAS, which is a 20-item questionnaire assessing difficulties recognizing and understanding emotions. Respondents answer using a five-point Likert scale (1 = *strongly disagree*; 5 = *strongly agree*); higher scores refer to higher levels of various aspects of alexithymia. Validation studies have established that the TAS-20 is a valid and reliable measure [58]; this also holds true for the Hungarian adaptation [59]. The TAS-20 consists of three subscales, but we will only report and test the subscale “Difficulty Identifying Feelings” (DIF; 7 items, Cronbach’s α = .79), as the other two subscales had very low internal consistencies in this study (Cronbach’s α = .20) and therefore would lead to invalid results.

#### Anxiety Sensitivity Index (ASI)

This instrument was created by Reiss and colleagues [60] to measure the predisposition of expecting negative consequences of experiencing anxiety. The respondents answer the 16 questions on a scale ranging from 0 (*very little*) to 4 (*very much*); higher scores indicate higher levels of anxiety sensitivity. Although there has been some debate about the factor structure, in total the ASI is a reliable and valid measure [61]. The Hungarian version used in this study [62] showed a good internal consistency of α = .87.

#### State Trait Anxiety Inventory–Trait Inventory (STAI-T)

Spielberger and colleagues [63] developed the STAI-T as a measure of trait anxiety. It consists of 20 questions which are answered on a scale of 0 (*almost never*) to 3 (*almost always*), with higher scores indicating more anxiety. The Hungarian version of the scale has good concurrent and construct validity [64]. In our sample, the internal consistency was α = .92.

#### Beck’s Depression Inventory (BDI-II)

The BDI [65] assesses common depressive symptoms using 21 questions. Answers are given on a 4-point Likert scale, with 1 indicating the symptom is not experienced and 4 indicating the highest possible symptom presence. Higher total scores refer to higher levels of depressive symptoms. As one of the most famous tools to measure depression, the BDI has been validated in many settings and languages; the short Hungarian version of the scale (BDI-9) consists of 9 items; it is characterised by good validity and reliability [66]. In the present study, its internal consistency was α = .81.

#### Patient Health Questionnaire Somatic Symptom Severity Scale (PHQ-15)

The PHQ-15 [67] is a 15-item instrument that assesses somatic symptom severity in the past 4 weeks. 13 of these items assess classical bodily symptoms (e.g., headaches, dizziness) and are scored on a scale from 0 (*not bothered at all*) to 2 (*bothered a lot*); the last two items are related to fatigue and associated with depression (e.g., trouble sleeping). Higher scores indicate more severe symptoms; clinical cutoff-points for low, moderate, and high symptom severity are scores of 5, 10, and 15, respectively [67]. The PHQ-15 is a reliable and valid tool for screening for somatization [67, 68]. In our sample, the Hungarian version [69] showed an internal consistency of α = .78.

#### Body Attitude Test (BAT)

Originally developed for female eating disorder patients, this questionnaire by Probst et al. [70] is designed to measure subjective experiences and attitudes towards one’s body. It consists of 20 questions which are answered on a 6-point Likert scale (0 = *never*, 5 = *always*). Three factors were identified: negative appreciation of body size, lack of familiarity with one’s own body, and general body dissatisfaction. In the present study, only the total score was used; higher total scores show more dissatisfaction. The Hungarian version’s [71] internal consistency was α = .86 for our sample.

#### Body Perception Questionnaire Body Awareness Scale (BPQ-BA-26)

The body perception questionnaire [72] was developed to measure various aspects of physical experiences pertaining to the body and stress responses. In this study, the short form of the body awareness scale (BPQ-BA-26) was used. Answer options for each question range from 1 (*never*) to 5 (*always*), higher scores indicate higher levels of body focus. The questionnaire is considered one of the first self-report instruments that assess interoceptive attention [73, 74], and is considered a reliable and valid measure of body focus [75]. The internal consistency of the Hungarian version [74] was α = .90.

#### Procedure

Questionnaires were completed on the day before the laboratory measurement. In the laboratory, participants read and signed an informed consent form. Their body composition was measured with an Omron BF511 body composition monitor (OMRON Healthcare Group, Kyoto, Japan), blood pressure was assessed with Omron BP7100 upper arm blood pressure monitor (OMRON Healthcare Group, Kyoto, Japan). Following the placement of ECG and EMG electrodes, mental and motor tasks were administered in a randomized order in a sitting position after a 3-minute-long resting period. In each task, participants received the instruction from an audio tape, then completed a 15 sec practice trial and three measurement trials (25, 35, and 50 sec in random order).

### Statistical analysis

Statistical analysis was carried out using the JASP v0.16.4 software [76]. Differences among CAc indices of the mental and motor tracking tasks were checked with repeated measures analysis of variance (ANOVA) with Greenhouse-Geisser correction; in the *post hoc* analysis, Holm correction was applied. Differences in CSb indices were calculated with Mann-Whitney test with rank-biserial correlation as indicator of ES. Association between indicators of CAc were estimated with Spearman correlation. For the calculations of circular statistics, the circ_r function of the Circular Statistics Matlab toolbox [57] was used. Significant deviation of angle series (calculated by the time differences between the R-peaks and the motor responses compared to the average RR time) from a uniform distribution was tested with the Rayleigh test; a *p*-value below the *p* = 0.05 limit indicates that the distribution of the finger movements with respect to the R-R peaks cannot be considered random; in other words, there is a temporal association between finger movements and the preceding heartbeat. Differences between detectors and non-detectors with respect to various indices of CAc/CSb were tested with Mann-Whitney test. Associations between indicators of CAc/CSb and the assessed questionnaire scores were tested with frequentist and Bayesian correlation analysis (Pearson correlation). Because of the high number of independent tests, accepted level of *p* was set to 0.002 for CAc and 0.004 for CSb. Finally, differences between detectors and non-detectors in terms of questionnaire scores were also checked with the frequentist and Bayesian version of independent samples t-test. In the Bayesian analysis, a Bayes Factor (BF_10_) below .33 was considered as supporting the null hypothesis (i.e., lack of association or difference), a BF_10_ above 3 indicated the superiority of the alternative hypothesis, and a BF_10_ in the middle domain (i.e., ranging from .33 to 3) was regarded as inconclusive [77].

## Results

### Cardioceptive accuracy

Descriptive statistics of the calculated indices are summarized in Table 1. The mean values of the indices calculated with the Schandry formula were relatively low for the mental tracking task, and even lower assessed with the motor task; on average, participants reported/indicated approximately every third (CAc_mental_), every fifth (CAc_motor_all_) or every twelfth (CAc_motor_acceptable_) heartbeat only. Also, mean value of the mean resultant vector indicated low temporal consistency of motor responses.

**Table 1:** Descriptive statistics of the assessed and calculated variables.

Index of cardioceptive accuracy (N = 102)	M	SD	min	max
CAc_mental_	0.37	0.241	0	0.95
CAc_motor_all_	0.20	0.194	0	0.91
CAc_motor_acceptable_	0.08	0.081	0	0.38
mean resultant vector	0.26	0.229	0	0.99

**Fig 1:**
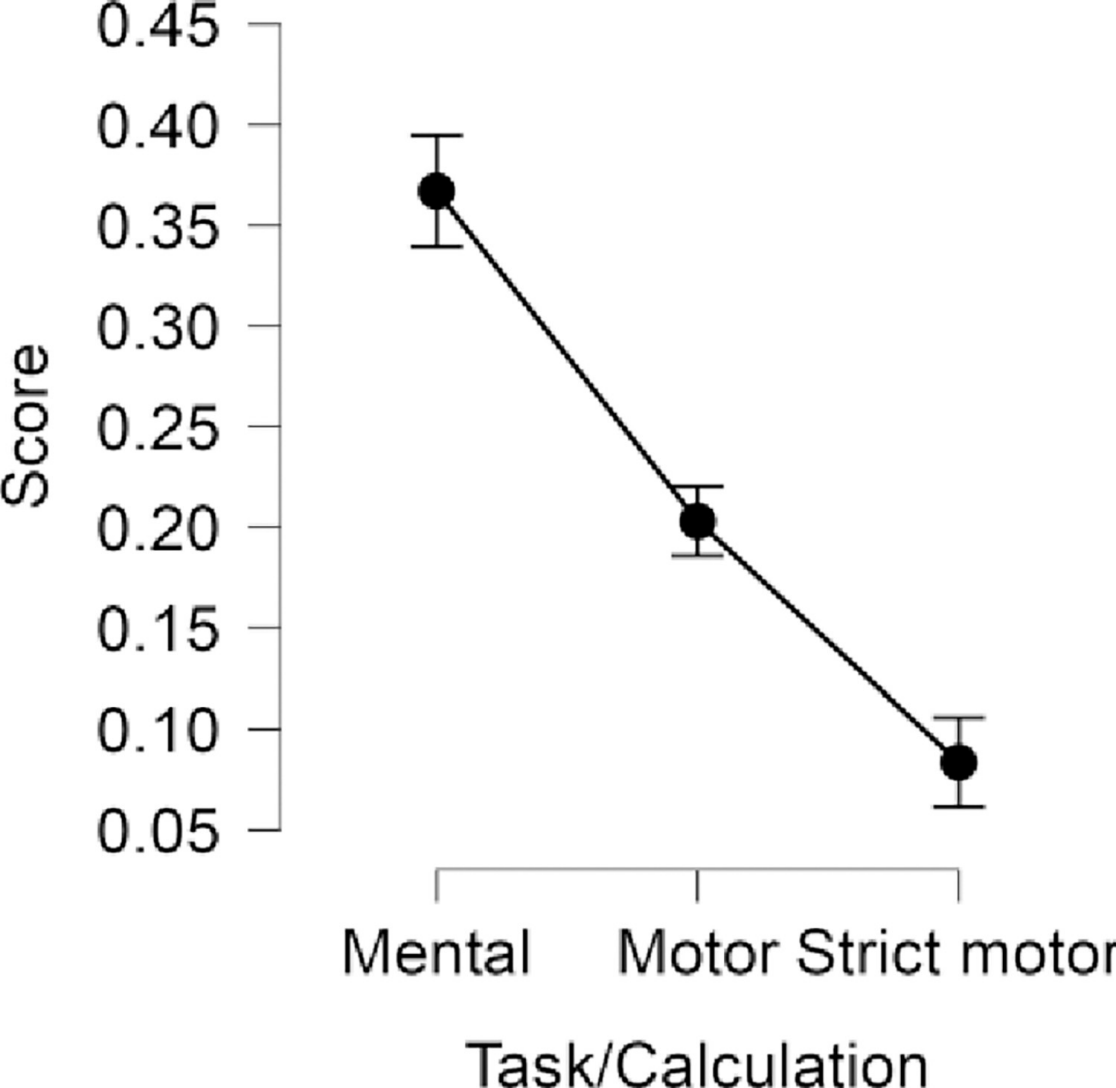
Average scores achieved by participants in the mental and motor tracking task. All differences are significant (p < .05), error bars indicate 95% confidence intervals.

Repeated measures ANOVA indicated significant differences between the indices (*F*(1.566,158.145) = 153.329, *p* < .001, η^2^ = 0.6034). *Post hoc* analysis showed significant (*p*_Holm_ < 0.05) differences between each pair for both conditions (Fig 1). Correlation analysis indicated strong associations between CAc_mental_, CAc_motor_all_, and CAc_motor_acceptable_ (Table 2). Mean resultant vector was not significantly associated with CAc_mental_;, i.e., higher Schandry scores were not associated with higher levels of consistency between heartbeats and motor responses. However, it was reversely related to CAc_motor_all_ and CAc_motor_acceptable_.

**Table 2:** Associations (Spearman correlations) between indicators of CAc.

n = 102	CAc_motor_all_	CAc_motor_acceptable_	mean resultant vector
CAc_mental_	0.72***	0.64***	-0.14
CAc_motor_all_		0.94***	-0.40***
CAc_motor_acceptable_			-0.44***

**Fig 2:**
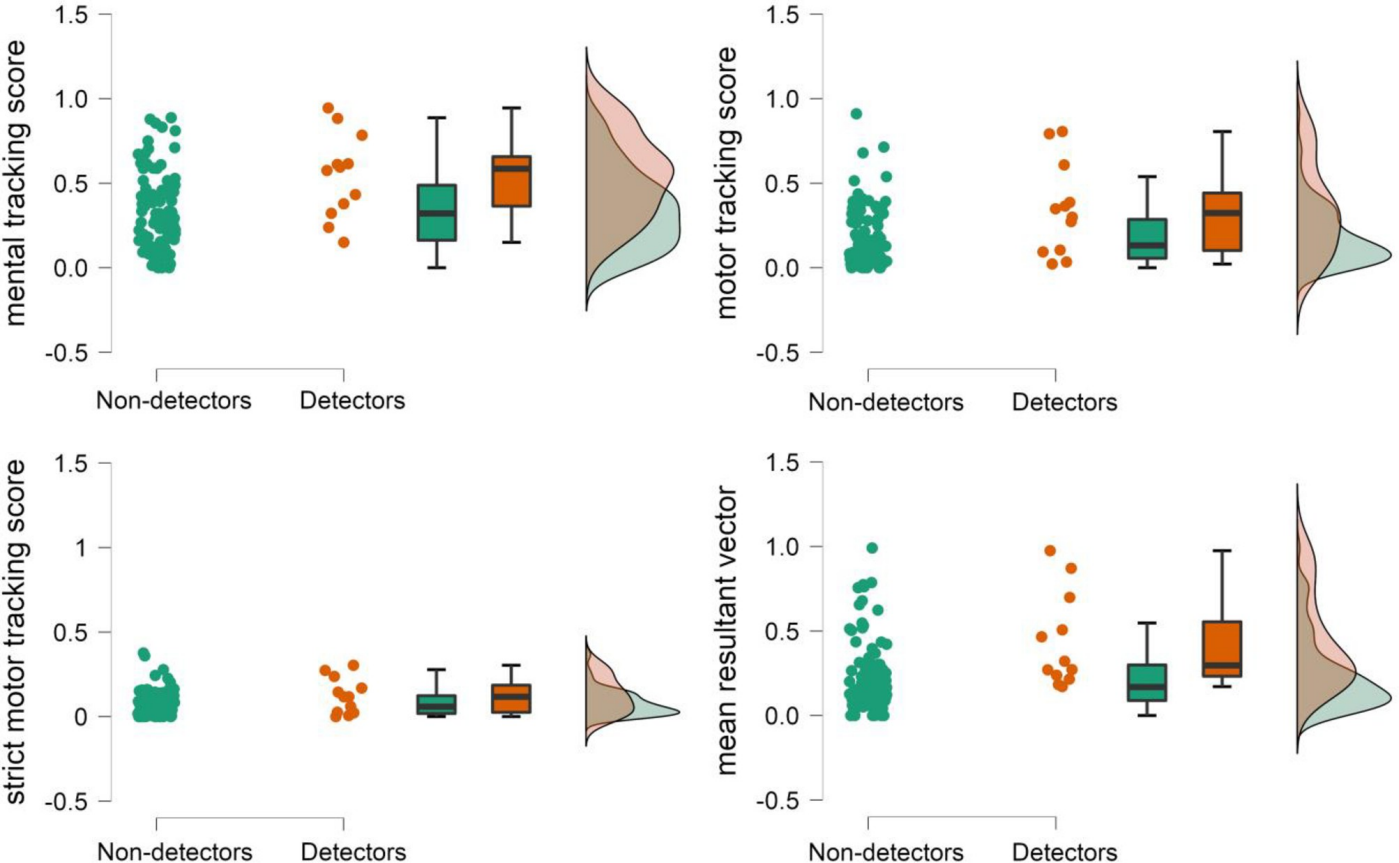
Distribution of various indicators of CAc for detectors and non-detectors.

According to the results of the Rayleigh test, motor responses of only 12 participants out of 102 were significantly (*p* < 0.05) associated with heartbeats. The difference between detectors and non-detectors was significant for CAc_mental_resting_, CAc_motor_resting_all_, and mean resultant vector (detectors uniformly showed higher mean values than non-detectors), but not for CAc_motor_resting_acceptable_ (for details, see Table 3 and Fig 2).

**Table 3:** Descriptive statistics (M±SD) of the detector and non-detector group (based on the temporal consistency of the motor responses), and outcome of Mann-Whitney test.

Index of cardioceptive accuracy	Detectors (N = 12)	Non-detectors (N = 90)	W	*p*	*r*_rank-biserial_
CAc_mental_resting_	0.54±0.249	0.34±0.231	299.000	0.012	0.446
CAc_motor_resting_all_	0.34±0.272	0.18±0.174	349.000	0.048	0.354
CAc_motor_resting_acceptable_	0.12±0.106	0.08±0.077	412.000	0.185	0.237
mean resultant vector	0.43±0.277	0.23±0.213	255.000	0.003	-0.528

### Cardioceptive sensibility (confidence ratings)

**Fig 3:**
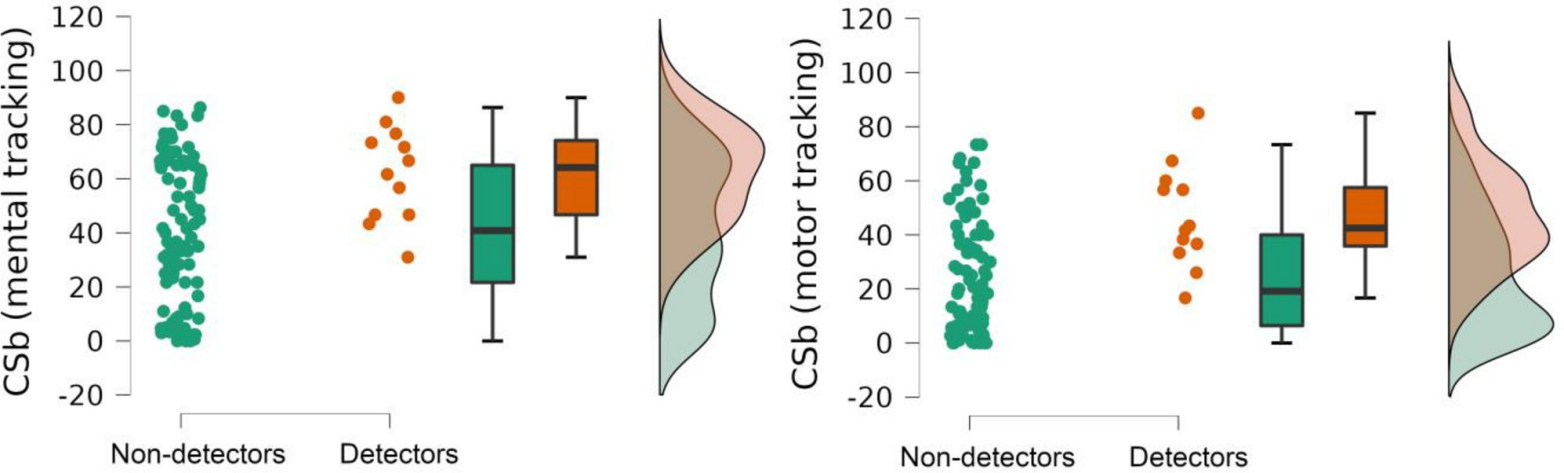
Distribution of indicators of CSb for detectors and non-detectors.

CSb with respect to the mental tracking task was significantly higher than that of the motor tracking task (M±SD: 43.53±26.28 and 27.28±21.96, respectively; *W* = 4407.000, *p* < .001, *r*_rank-biserial_ = 0.116). Detectors showed significantly higher values than for non-detectors for both Csb_mental_ (M±SD: 62.11±17.61 and 41.06±26.33, respectively; *W* = 291.500, *p* = .01, *r*_rank-biserial_ = 0.176) and CSb_motor_ (M±SD: 46.81±19.03 and 24.68±21.08, respectively; *W* = 234.500, *p* = .002, *r*_rank-biserial_ = 0.176) (Fig 3).

### Associations with questionnaires

Descriptive statistics of the assessed questionnaires are presented in Table 4. Note that a substantial level of variance can be found for all variables; in other words, our sample was heterogeneous with respect to these constructs.

**Table 4:** Descriptive statistics of the assessed questionnaires.

n = 102	M	SD	min	max
TAS DIF	17.6	5.16	8	32
ASI	25.3	12.86	6	58
STAI-T	26.3	10.36	5	54
BDI-9	15.4	5.76	9	34
PHQ-15	7.9	4.74	0	21
BPQ-BA-26	88.4	16.67	38	124
BAT	39.6	15.83	0	86

Note. Abbr.: TAS DIF: Toronto Alexithymia Scale Difficulty Identifying Feeling subscale; ASI: Anxiety Sensitivity Index; STAI-T: State-Trait Anxiety Inventory Trait Inventory; BDI-9: Beck Depression Inventory 9-item version; PHQ-15: Patient Health Questionnaire Somatic Symptom Severity Scale; BPQ-BA-26: Body Perception Questionnaire Body Awareness Scale; BAT: Body Attitude Test

Frequentist correlation analysis did not show significant associations between indicators of CAc and questionnaire scores at the level of *p* < 0.002 (Table 5; for scatter plots, see S1 and S2 Figs). Typically, correlation coefficients were below 0.1, indicating very weak effects. In line with this, Bayesian analysis supported the superiority of null hypothesis (lack of association; BF_10_ < .33) for the vast majority of the cases and was inconclusive (0.33 < BF_10_ < 3) for the rest of correlations (Table 5). Overall, it can be concluded that CAc is independent from the assessed constructs.

**Table 5:** Associations between indicators of CAc and self-reported characteristics. Results of frequentist and Bayesian correlation analysis.

n = 102	CAc_mental_resting_			CAc_motor_resting_all_			CAc_motor_resting_acceptable_			mean resultant vector		
	*r*	*p*	*BF*_*10*_	*r*_*s*_	*p*	*BF*_*10*_	*r*_*s*_	*p*	*BF*_*10*_	*r*_*s*_	*p*	*BF*_*10*_
TAS DIF	0.164	0.099	0.475	0.055	0.584	0.143	0.082	0.413	0.172	-0.027	0.785	0.128
ASI	0.075	0.451	0.164	0.028	0.777	0.129	0.065	0.517	0.152	-0.002	0.987	0.124
STAI-T	0.097	0.334	0.196	0.049	0.624	0.139	0.091	0.363	0.186	-0.006	0.951	0.124
BDI-9	0.071	0.477	0.159	0.011	0.911	0.125	0.030	0.765	0.129	0.084	0.402	0.175
PHQ-15	0.171	0.085	0.533	0.077	0.442	0.166	0.079	0.431	0.168	-0.082	0.415	0.172
BPQ-BA-26	-0.016	0.872	0.125	-0.027	0.784	0.128	0.037	0.713	0.132	0.106	0.290	0.215
BAT	0.175	0.078	0.571	0.102	0.307	0.207	0.124	0.215	0.264	-0.156	0.117	0.416

Note. Abbr.: TAS DIF: Toronto Alexithymia Scale Difficulty Identifying Feeling subscale; ASI: Anxiety Sensitivity Index; STAI-T: State-Trait Anxiety Inventory Trait Inventory; BDI-9: Beck Depression Inventory 9-item version; PHQ-15: Patient Health Questionnaire Somatic Symptom Severity Scale; BPQ-BA-26: Body Perception Questionnaire Body Awareness Scale; BAT: Body Attitude Test

No significant differences between detectors and non-detectors were found in any assessed variables; again, effect sizes typically were in the weak domain. Bayesian analysis supported the null hypothesis for most of the cases, or was inconsistent (for details, see Table 6).

**Table 6:** Comparison of detectors and non-detectors with respect to the assessed self-report variables.

	M±SD Detectors (n = 12)		M±SD Non-detectors (n = 90)		t(100)	*p*	*d*	*BF*_*10*_
TAS DIF	18.333	6.272	17.533	5.028	-0.503	0.616	-0.154	0.334
ASI	25.667	16.267	25.300	12.444	-0.092	0.927	-0.028	0.303
STAI-T	25.583	13.104	26.433	10.026	0.266	0.791	0.082	0.311
BDI-9	16.333	7.820	15.300	5.478	-0.581	0.562	-0.179	0.345
PHQ-15	8.417	6.186	7.800	4.552	-0.422	0.674	-0.130	0.324
BPQ-BA-26	86.667	23.043	88.644	15.786	0.384	0.701	0.118	0.320
BAT	35.833	24.698	40.122	14.378	0.881	0.381	0.271	0.411

Note. Abbr.: TAS DIF: Toronto Alexithymia Scale Difficulty Identifying Feeling subscale; ASI: Anxiety Sensitivity Index; STAI-T: State-Trait Anxiety Inventory Trait Inventory; BDI-9: Beck Depression Inventory 9-item version; PHQ-15: Patient Health Questionnaire Somatic Symptom Severity Scale; BPQ-BA-26: Body Perception Questionnaire Body Awareness Scale; BAT: Body Attitude Test

Frequentist and Bayesian correlation analyses did not indicate any significant associations between CSb in the two tasks and questionnaire scores (Table 7). In fact, the superiority of the null hypothesis was supported in all cases but one.

**Table 7:** Associations between indicators of CSb and self-reported characteristics. Results of frequentist and Bayesian correlation analysis.

n = 102	CSb_mental_			CSb_motor_		
	*r*	*p*	*BF*_*10*_	*r*_*s*_	*p*	*BF*_*10*_
TAS DIF	0.113	0.260	0.231	0.018	0.855	0.126
ASI	0.118	0.236	0.247	0.069	0.492	0.156
STAI-T	0.027	0.785	0.128	-0.032	0.749	0.130
BDI-9	0.009	0.932	0.124	-0.090	0.369	0.184
PHQ-15	0.149	0.135	0.373	0.034	0.733	0.131
BPQ-BA-26	0.055	0.580	0.144	0.002	0.980	0.124
BAT	0.100	0.316	0.203	-0.006	0.949	0.124

Note. Abbr.: TAS DIF: Toronto Alexithymia Scale Difficulty Identifying Feeling subscale; ASI: Anxiety Sensitivity Index; STAI-T: State-Trait Anxiety Inventory Trait Inventory; BDI-9: Beck Depression Inventory 9-item version; PHQ-15: Patient Health Questionnaire Somatic Symptom Severity Scale; BPQ-BA-26: Body Perception Questionnaire Body Awareness Scale; BAT: Body Attitude Test

## Discussion

In a laboratory study with 102 young individuals, a strong association between performance in the mental heartbeat tracking task and a novel motor tracking task was found. However, the temporal consistency between motor responses and preceding heartbeats showed a moderate negative association with motor tracking performance; moreover, it was not significantly associated with mental tracking performance. Finally, results of frequentist and Bayesian analysis suggest the lack of association between any indices of cardioceptive accuracy and anxiety sensitivity, trait anxiety, depression, somatic symptom distress, body awareness, body image dissatisfaction, and alexithymia.

Concerning the strong positive associations between mental tracking score and motor tracking scores, findings of our previous study [18] were replicated in a larger sample. Also, the mean values of CAc_mental_ (0.37±0.24 *vs* 0.38±0.28), CAc_motor_all_ (0.20±0.19 *vs* 0.25±0.23), and CAc_motor_acceptable_ (0.08±0.08 *vs* 0.10±.010) were comparable to those of the previous study. These results suggest that, although even detectors’ ability to perceive heartbeats is generally poor, not to speak of non-detectors, the mental tracking task does have a bottom-up (interoceptive) component [15, 18, 55]. Inclusion of the mean resultant vector, an indicator of consistency between heartbeats and motor responses, in the analysis somewhat modifies this conclusion. Those with higher consistency showed fewer motor responses, which might be the consequence of interference between cardiac and proprioceptive (motor response related) sensations. Alternatively, it can be the result of temporal or other limitations of attentional processes; quick periodic switches between perception of cardiac sensations and initiation of motor responses may be overly demanding in the long run. However, those with a better synchrony between motor responses and cardiac events in the motor task were not characterized by a higher mental tracking score. This suggests that the motor and mental tracking tasks rely on partly different cognitive processes; for example the decision criterion might be different in the two tasks. Still, as the performance of detectors and non-detectors significantly differed with respect to both CAc_mental_ and CAc_motor_all_, it cannot be stated that the mental tracking task completely lacks a bottom-up (sensory) component. Surprisingly, no significant difference between detectors and non-detectors was found for CAc_motor_resting_acceptable_. It is possible that the time frame we used as acceptable (from 350ms to 650ms) was not appropriate for everyone thus a proportion of motor responses were not included in the calculation of the index. Indeed, previous research suggests that there are substantial individual differences in the delay between the R-peak of ECG and motor responses [78].

Similar to our previous study, only a minority of our participants (appr. 12%) could be considered detectors and even their cardiac accuracy was poor. Overall, this supports the idea that the ability to accurately perceive heartbeats is not necessary for normal psychological functioning; in fact, the lack of this ability characterized the majority of the sample. In line with this conclusion, a number of authors proposed that, considering the limitations of human stimulus processing capacity, it is more adaptive to focus on external rather than interoceptive cues [79–81]. As perception of heartbeats is not needed for physiological regulation of the cardiovascular system, it is possible that it is simply a by-product (i.e., a neutral feature) of the rhythmic movements of the heart [3]. As the cardiac signal is weak and not salient, we learn to ignore it under everyday circumstances [79]. In consequence, when participants are asked to try to sense it in the laboratory, they necessarily use top-down cues (“perceptual heuristics”) to perceive or estimate it [18, 82].

The speculation that perception of heartbeat is not a meaningful or adaptive feature receives further support from the self-report related findings of the study. This is the first study that applies Bayesian analysis to the estimation of association between CAc and a wide range of trait-like self-report variables. This way, we were able to not only reject the alternative hypotheses but to calculate the relative probabilities of the null and the alternative hypotheses. These probabilities indicated the superiority of the null hypotheses for most cases. This is in line with the findings of a recent meta-analysis, reporting non-significant associations between CAc and trait anxiety, depression, and alexithymia [33], and the lack of significant associations between self-reported interoception and CAc [41, 43, 45, 46]. The negative association between body image dissatisfaction and cardioceptive accuracy reported in several studies [40–42] was not supported by our present findings. It is important to note that the above cited studies applied the Schandry task with a relaxed instruction which favours top-down factors in the perception of heartbeats [19]. In contrast to our findings and more recent meta-analyses [32, 33], a meta-analysis indicated a positive association with medium to large effect size (d = 0.63) between anxiety sensitivity and CAc [31]. However, methodological flaws were reported for some of the included studies [31]; in addition, the use of a strict instruction that explicitly prohibits estimation was not a typical practice at the time. Overall, the lack of associations between CAc and a wide range of trait-like characteristics that can heavily impact our everyday functioning, such as anxiety, anxiety sensitivity, depression, somatic symptom distress, body awareness, body image dissatisfaction, and alexithymia, is in accordance with the assumed unimportance of heartbeat perception ability.

Beyond these findings, our results support the lack of association between indicators of CSb, meaning perceived performance or confidence in the tasks, and questionnaire scores. CSb showed no significant association with anxiety in a study [83]; however, nothing is known about its relation to other self-report measures. The non-existing association between CSb and body awareness [2], a trait-like construct that generalizes body focus and perceived awareness of internal signals across modalities over time [74], is particularly striking [84]. These findings fit very well the ideas raised above. If one takes into consideration the fact that heartbeats are very vague stimuli that are rarely sensed, it appears logical that perceived ability might contribute to self-reports rather than actual ability. As this is not the case, it can be concluded that perception of heartbeat, regardless of its bottom-up (physiological) or top-down origin, is not associated with the assessed constructs.

Participants of the study were young individuals, not representing the general population. This limits the generalizability of our findings, particularly to clinical populations. Although range of the questionnaire scores was quite wide for all constructs, replication of the study in respective clinical populations would shed more light on the associations between CAc and various mental health problems. Moreover, low scores in the motor tracking task can partly be due to interference between motor responses and perception of heartbeats.

## Conclusion

The ability to accurately perceive heartbeats is poor even under resting conditions, in the absence of distracting external cues. Cardioceptive accuracy is not associated with trait anxiety, anxiety sensitivity, depression, somatic symptom distress, body awareness, body image dissatisfaction, and alexithymia in young individuals.

## Supporting information

S1 File Instructions of the tracking tasks. (DOC)

S1 Fig Scatter plots of the correlation analyses, No.1. Note. Abbr.: TAS DIF: Toronto Alexithymia Scale Difficulty Identifying Feeling subscale; ASI: Anxiety Sensitivity Index; STAI-T: State-Trait Anxiety Inventory Trait Inventory; BDI-9: Beck Depression Inventory 9-item version.
(TIF)

S2 Fig Scatter plots of the correlation analyses, No.2. Note. Abbr.: PHQ-15: Patient Health Questionnaire Somatic Symptom Severity Scale; BPQ-BA-26: Body Perception Questionnaire Body Awareness Scale; BAT: Body Attitude Test.
(TIF)
