# Supplementary material for: Do we need to accurately perceive our heartbeats? Cardioceptive accuracy and sensibility are independent from indicators of negative affectivity, body awareness, body image dissatisfaction, and alexithymia
Source: PLoS One. 2023 Jul 5;18(7):e0287898. doi: 10.1371/journal.pone.0287898 (PMC10321613; doi:10.1371/journal.pone.0287898)
Supplement: S1 File — (DOC) [file pone.0287898.s001.doc]

Mental tracking task

Please, sit comfortably, with both feet on the ground, hands on thighs. I will ask you to count your heartbeats. How readily we can sense our heartbeats varies. You have surely noticed that after running up the stairs you are able to count your heartbeat precisely; other times, you are not aware of them at all. Now we are interested in how you can sense them here and now. It is important that you must count only the sensed heartbeats, and please, avoid guessing. However, if you have a slight sensation, you can count it. If you do not feel anything, please, feel free to say zero. Please, start counting when I say NOW; and when I say STOP, please, report how many heart beats you counted. Please, keep your eyes open during the task. First, there will be a 15 sec long practice session, then three sessions of varying length. Do you have any questions before we start?

Motor tracking task

Please, sit comfortably, with both feet on the ground, hands on thighs. The palm of your right hand should be upward. I will ask you to focus on your heartbeats, and every time when you sense one, right away and firmly move your right index finger toward your palm. How readily we can sense our heartbeats varies. You have surely noticed that after running up the stairs you are able to count your heartbeat precisely; other times, you are not aware of them at all. Now we are interested in how you can sense them here and now. It is important that you must move your finger only if you actually sense a heartbeat, and please, avoid guessing. However, if you have a slight sensation, you can indicate it. If you do not feel anything, please, never move your finger. Please, start when I say NOW; and when I say STOP, please, don’t move you index finger anymore. Please, keep your eyes open during the task. First, there will be a 15 sec long practice session, then three sessions of varying length. Do you have any questions before we start?
