# Supplementary figures and images for: Do we need to accurately perceive our heartbeats? Cardioceptive accuracy and sensibility are independent from indicators of negative affectivity, body awareness, body image dissatisfaction, and alexithymia
Source: PLoS One. 2023 Jul 5;18(7):e0287898. doi: 10.1371/journal.pone.0287898 (PMC10321613; doi:10.1371/journal.pone.0287898)

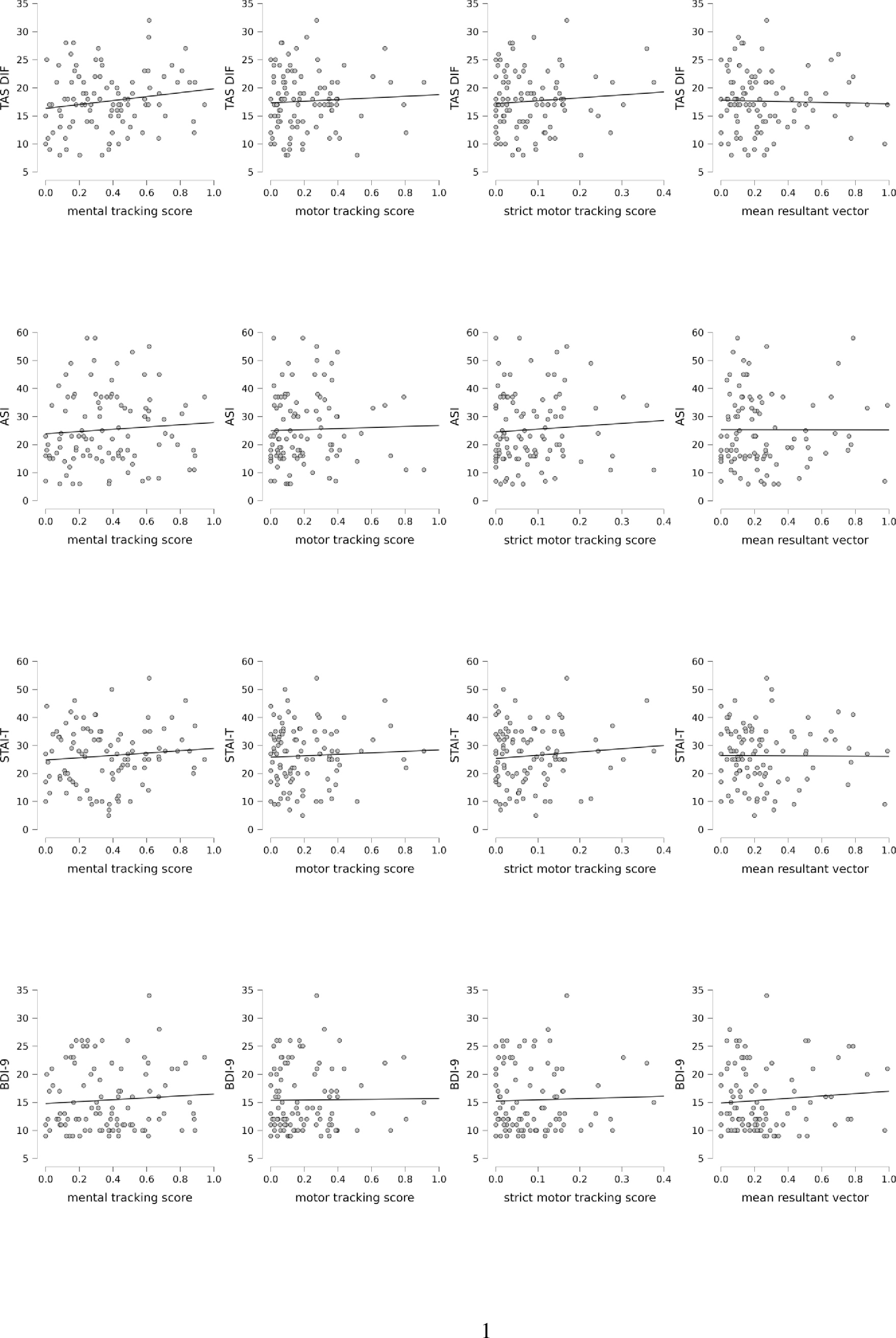

Supplement: S1 Fig — Note. Abbr.: TAS DIF: Toronto Alexithymia Scale Difficulty Identifying Feeling subscale; ASI: Anxiety Sensitivity Index; STAI-T: State-Trait Anxiety Inventory Trait Inventory; BDI-9: Beck Depression Inventory 9-item version. (TIF) [file pone.0287898.s002.tif]

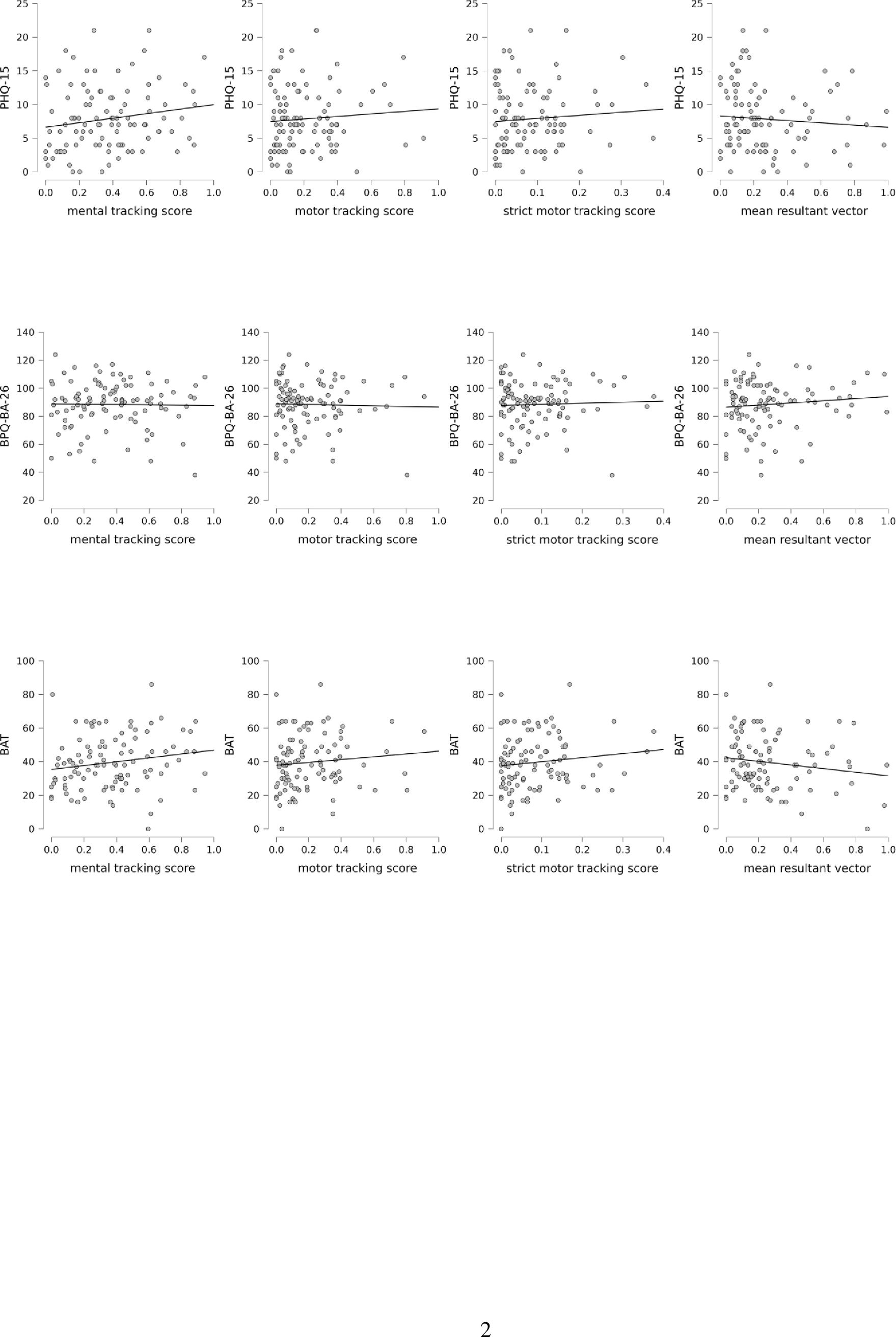

Supplement: S2 Fig — Note. Abbr.: PHQ-15: Patient Health Questionnaire Somatic Symptom Severity Scale; BPQ-BA-26: Body Perception Questionnaire Body Awareness Scale; BAT: Body Attitude Test. (TIF) [file pone.0287898.s003.tif]
